# Associations between sleep duration, sleep quality and diabetic retinopathy

**DOI:** 10.1371/journal.pone.0196399

**Published:** 2018-05-24

**Authors:** Nicholas Y. Q. Tan, Merwyn Chew, Yih-Chung Tham, Quang Duc Nguyen, Masayuki Yasuda, Ching-Yu Cheng, Tien Yin Wong, Charumathi Sabanayagam

**Affiliations:** 1 Singapore Eye Research Institute and Singapore National Eye Centre, Singapore; 2 Department of Ophthalmology, National University Hospital, Singapore; 3 Department of Ophthalmology, Yong Loo Lin School of Medicine, National University of Singapore, Singapore; 4 Ophthalmology and Visual Sciences Academic Clinical Program, Duke-NUS Medical School, National University of Singapore, Singapore; Soochow University Medical College, CHINA

## Abstract

**Background:**

Abnormal durations of sleep have been associated with risk of diabetes. However, it is not clear if sleep duration is associated with diabetic retinopathy (DR).

**Methods:**

In a cross-sectional study, we included 1,231 (Malay, *n* = 395; Indian, *n* = 836) adults (mean age 64.4 ± 9.0 years, 50.4% female) with diabetes from the second visit of two independent population-based cohort studies (2011–15) in Singapore. Self-reported habitual sleep duration was categorized as short (<6 h), normal (6≤ h <8), and long (≥8 h). Questionnaires were administered to detect risk of obstructive sleep apnea (OSA), excessive daytime sleepiness, and insomnia, all of which may indicate poor quality of sleep. The associations between sleep-related characteristics with moderate DR and vision-threatening DR (VTDR) were analysed using logistic regression models adjusted for potential confounders.

**Results:**

Prevalence of moderate DR and VTDR in the study population were 10.5% and 6.3% respectively. The mean duration of sleep was 6.4 ± 1.5 h. Compared to normal sleep duration, both short and long sleep durations were associated with moderate DR with multivariable odds ratio (95% confidence interval) of 1.73 (1.03–2.89) and 2.17 (1.28–3.66) respectively. Long sleep duration (2.37 [1.16–4.89]), high risk of OSA (2.24 [1.09–4.75]), and excessive daytime sleepiness (3.27 [1.02–10.30]) were separately associated with VTDR.

**Conclusion:**

Sleep duration had a U-shaped association with moderate DR; long sleep duration, excessive daytime sleepiness and high risk of OSA were positively associated with VTDR.

## Introduction

Diabetic retinopathy (DR) is a common and major complication of diabetes, and is the leading cause of preventable blindness in working-age adults [1]. The prevention and treatment of DR is a growing public health challenge, given the growing prevalence of diabetes, and the substantial burden of DR worldwide [2]. Thus, it is important from both clinical and public health perspectives, to identify other modifiable risk factors in addition to the classic risk factors such as poor glycemic control, hypertension, and longer duration of disease [3].

Sleep is a complex and highly organised biological and behavioural process that serves many functions, and when deficient or disorganised, may result in ill-health and increased overall mortality [4]. An increasing prevalence of poor sleep health, which shows secular trends alongside changes in modern society, is an underappreciated determinant of health status. Recently, a growing body of evidence from both experimental and epidemiological studies have shown sleep duration and sleep quality (for instance, in those with sleep breathing disorders) to be significantly associated with diabetes, insulin resistance, and poor glycemic control [5–8].

Considering the close association between diabetes and DR, it is reasonable to hypothesise that sleep may also have associations with DR. However, only three cross-sectional studies have investigated the relationship between sleep duration and DR thus far [9–11], of which only one has examined for severity of DR. Furthermore, whether obstructive sleep apnea (OSA) may predispose towards DR is still up for debate, as results have not always been consistent [12]. To address these gaps, we examined the association of sleep duration and sleep quality with the presence and severity of DR in Asian adults with diabetes who attended the Singapore Malay Eye Study (SiMES) and the Singapore Indian Eye Study (SINDI).

## Methods

### Study population

SiMES (2004–07, *n* = 3280) and SINDI (2007–09, *n* = 3400) are two independent population-based cohort studies of Malay and Indian adults in Singapore. Follow-up visits were conducted from 2011–2015. The detailed methodology of SiMES-2 [13] and SINDI-2 [14] have been reported elsewhere. In brief, 1,901 Malay participants (72.1% of eligible participants) and 2,200 Indian participants (75.5% of eligible participants) attended the SiMES-2 and SINDI-2 studies respectively. Sleep-related data were collected only in the follow-up studies and not at baseline. Hence, for the current study, we used the cross-sectional data from the follow-up visit. Both studies followed similar methodology and were conducted in the same study clinic. Written informed consent was obtained from all participants. The study adhered to the tenets of the declaration of Helsinki, and ethics committee approval was obtained from the SingHealth Centralised Institutional Review Board. Since the methodology was similar, for the present analysis, we combined both study participants.

Of the 4,101 participants, 1,587 (38.7%) had diabetes (n = 642 Malays and 945 Indians). Of these, 1,385 participants completed all the questionnaires on sleep health included in our study protocol. We excluded a further 41 participants who did not have gradable fundus photographs, and 113 participants who had missing data on other key variables used in the multivariable models. Therefore, a total of 1,231 participants (*n* = 395 Malay, 836 Indian) were included in our analyses.

### Assessment of diabetic retinopathy

DR was assessed from retinal photographs according to a standardized protocol [15]. After pupil dilation, two retinal images of each eye were obtained using a digital retinal camera (Canon CR-DGi with a 10-D SLR back; Canon, Tokyo, Japan): one centred on the optic disc (Early Treatment for DR Study (ETDRS) standard field 1); and the other centred on the fovea (ETDRS standard field 2). Trained graders analysed the images for qualitative changes of DR. Retinopathy was considered present if any characteristic lesion as defined by the ETDRS severity scale was present: microaneurysms, hemorrhages, cotton wool spots, intraretinal microvascular abnormalities, hard exudates, venous bleeding, and new vessels [15]. For each eye, a retinopathy severity score was assigned according to the modified Airlie House Classification System: minimal nonproliferative DR (NPDR; levels 15–20), mild NPDR (level 35), moderate NPDR (levels 43–47), severe NPDR (level 53), and proliferative DR (PDR, levels >60) [16]. Disease severity was based on the eye with more severe DR changes. For our main analysis, we selected “moderate DR”, corresponding to a level of 43 and above, as the primary outcome. This level of retinopathy was selected because it was felt to be more diabetes-specific [17], and was used previously to derive the current diagnostic thresholds for diabetes. It was previously reported that mild levels of retinopathy are found in up to 10% of the general population without diabetes, but with hypertension (which is more common in sleep disorders) [18]. As a secondary outcome, we selected for “VTDR”, defined as the presence of severe NPDR, PDR, or clinically significant macular edema according to the Eye Diseases Prevalence Research Group definition [19].

### Sleep-related questionnaires and interviews

A standardized interviewer-administered questionnaire was conducted in English, Malay or Tamil, depending on the participant’s preference, to collect data on sleep-related variables. Self-reported habitual sleep duration was elicited from participants using the question: “on average, how much sleep do you get in a 24-hour day?” Participants responded using half-hour increments, and the duration of sleep was organised into 3 categories: short (<6 h), normal (6≤ h <8), and long (≥8 h). Validated questionnaires related to the quality of sleep were also verbally-administered to participants, which included: the Epworth Sleepiness Scale (ESS), the STOP-Bang questionnaire, and the Insomnia Severity Index (ISI).

Excessive daytime sleepiness is one of the most common symptoms of OSA; however, excessive daytime sleepiness also has other causes such as primary hypersomnias and secondary insomnias [20, 21]. The ESS is widely used as a subjective measure of the general level of daytime sleepiness [22]. The test determines the likelihood of falling asleep in eight daily situations (scores 0–3; total possible score of 24, where higher scores indicate greater sleepiness). Following the suggestion of the developer of this scale, a score ≥11 was classified as “excessive daytime sleepiness” [22].

The STOP-Bang questionnaire is a screening tool for OSA [23], which in clinical or subclinical forms, may often cause poor sleep quality. It consists of 4 questions related to snoring, tiredness, observed apneas, and high blood pressure, along with 4 additional demographic questions that include BMI, age, neck circumference, and gender. “High risk for OSA” was defined as ≥ 3 positive answers to the 8 STOP-Bang items [23].

The Insomnia Severity Index is a questionnaire that indicates the subjective global severity of insomnia, including perceived daytime consequences and distress [24]. The score range is between 0–28 for the 7 items in total. Scores 0–7 represent “no clinically significant insomnia”, and scores 8–14, 15–21 and 22–28 represent “sub-threshold insomnia”, “moderate severity insomnia”, and severe clinical insomnia” respectively [24]. For the purposes of this study, due to the low prevalence of scores in the 15–21 (2.5%) and 22–28 (0.1%) range, scores ≥8 were defined as “high risk of insomnia”.

In addition, other interviewer-administered questionnaires were used to collect data on demographics (e.g., age, gender, ethnicity), level of education, medical history (e.g., history of respiratory disorders such as asthma and chronic obstructive pulmonary disease; use of insulin injections or anti-hypertensive medication), lifestyle factors (e.g., current cigarette smoking), and mood-related complaints [13, 14]. Educational level was recorded as the highest number of years of schooling completed and was categorized into 2 groups: (1) primary school level or lower (≤6 years), and (2) secondary school level or higher (≥7 years). Mood-related complaints were assessed using the question: “are you anxious or depressed?” Participants who reported being at least moderately anxious or depressed were considered to have mood-related complaints.

### Other assessments

Blood pressure was measured using a digital automatic blood pressure monitor (Dinamap model Pro100V2; Criticon GmbH, Norderstedt, Germany). Height was measured using a wall-mounted tape, and weight with a digital scale (SECA, model 782 2321009; Vogel & Halke, Hamburg, Germany). Body-mass index (BMI) was defined as weight divided by the square of height in meters (kg/m^2^). Obesity was defined as BMI of ≥27.5 kg/m^2^ according to the recommended WHO Asian BMI cut-off points [25]. Venous blood samples were collected to measure for random plasma glucose, haemoglobin A1c (HbA1c), serum cholesterol, and creatinine levels. Diabetes was defined as random plasma glucose of 200 mg/dl (11.1 mmol/l) or more, HbA1c of 6.5% or more, self-reported use of diabetic medication, or physician-diagnosed diabetes [26].

### Statistical analysis

Statistical analysis was performed using R 3.3.1 statistical computing language (R Core Team, 2016). The Kruskal-Wallis H Test (for categorical variables) and ANOVA (for continuous variables) were used to compare the demographic and clinical characteristics of participants between the three categories of sleep duration. Logistic regression models were used to assess the associations of sleep-related characteristics with moderate DR (the presence of moderate DR amongst participants with DM) in the primary analysis and with the severity of DR (the presence of VTDR amongst participants with any DR) in a secondary analysis. Sleep duration was treated as a categorical variable where short and long sleep durations were referenced against normal durations of sleep. Scores from the three sleep-related questionnaires were analysed as both categorical or continuous variables. In a first logistic regression model, odds ratios (ORs) were adjusted for age, gender and ethnicity; in a second model, additional adjustments were made for HbA1c, blood pressure, and other potential confounders such as antihypertensive medication, current smoking, respiratory disorders, mood-related complaints, level of education, obesity, and cholesterol levels. In a third model, in addition to variables adjusted for in the second model, we also included duration of diabetes and the use of insulin injections; cases with missing data on either of these two variables were excluded from this analysis. Additionally, to examine the dose-response relationship between sleep duration and moderate DR (and VTDR) without linearity assumptions, we used flexible nonparametric logistic regression employing the generalised additive modelling approach to calculate odds of moderate DR (and VTDR), adjusting for all covariates in the multivariable model 3; the predicted odds of moderate DR (and VTDR) were then plotted against increasing hours of sleep duration. *P* value for significance was set at <0.05.

## Results

A total of 1,231 participants with diabetes were included in the analyses; 395 (32.1%) were Malay and 836 (67.9%) were Indian; the mean age was 64.4 ± 9.0 years, and 621 (50.4%) were female. Of these, 129 (10.5%) had moderate DR and 77 (6.3%) had VTDR. The mean (SD) duration of sleep was 6.4 (1.5) h; 23.9%, 54.9%, and 21.2% participants had short, normal, and long durations of sleep respectively. The demographic and clinical characteristics of the study participants stratified by duration of sleep are presented in Table 1. Participants with normal duration of sleep were on average younger, more often had above primary school education, and were less often on insulin compared to those with short or long durations of sleep. Participants with short sleep duration showed a higher proportion of females, more respiratory disorders, more frequent mood-related complaints, higher ISI scores, and higher risk of insomnia, compared to those with normal or long sleep durations. There was no significant difference in the sleep duration between the two ethnicities.

**Table 1:** Demographic and clinical characteristics by sleep duration.

	Sleep duration			
	<6 h (*n* = 294)	6≤ h <8 (*n* = 676)	≥8 h (*n* = 261)	P value
**Characteristics**				
Age, years	64.7 (8.9)	63.8 (8.8)	65.4 (9.7)	0.04
Gender, Female, %	171 (58.2)	323 (47.8)	127 (48.7)	0.01
Ethnicity, Malay, %	98 (33.3)	227 (33.6)	70 (26.8)	0.12
Above primary school education, %	109 (37.1)	294 (43.5)	93 (35.6)	0.04
Current smoking, %	34 (11.6)	82 (12.1)	31 (11.9)	0.97
Duration of diabetes†, years	13.1 (9.3)	11.7 (8.4)	12.7 (9.6)	0.09
Insulin use‡, %	46 (19.3)	71 (12.7)	37 (18.0)	0.03
Hypertensive medication use, %	180 (61.2)	411 (60.8)	155 (59.4)	0.90
Mood-related complaints, %	126 (42.9)	220 (32.5)	73 (28.0)	0.001
Respiratory disorder, %	30 (10.2)	38 (5.6)	10 (3.8)	0.01
Systolic blood pressure, mmHg	139.9 (18.3)	139.4 (18.0)	141.9 (20.7)	0.18
Diastolic blood pressure, mmHg	75.5 (9.4)	75.7 (9.5)	76.2 (10.3)	0.68
Body-mass index, kg/m^2^	27.5 (4.9)	27.2 (4.7)	27.1 (5.1)	0.52
Obesity, %	200 (68.0)	466 (68.9)	183 (70.1)	0.87
Total cholesterol, mmol/L	4.89 (1.17)	4.77 (1.23)	4.86 (1.31)	0.33
LDL cholesterol, mmol/L	3.10 (0.94)	3.05 (1.00)	3.13 (1.08)	0.48
HbA1c, %	7.71 (1.64)	7.55 (1.49)	7.70 (1.58)	0.22
ESS score	3.9 (4.0)	4.1 (3.8)	3.9 (3.6)	0.69
Excessive daytime sleepiness, %	24 (8.2)	45 (6.7)	14 (5.4)	0.42
STOP-Bang score	2.6 (1.3)	2.6 (1.1)	2.6 (1.2)	0.74
High risk for OSA, %	141 (48.0)	339 (50.2)	129 (49.4)	0.82
ISI score	6.9 (5.2)	3.2 (3.3)	2.1 (2.9)	<0.001
High risk for insomnia, %	114 (38.8)	60 (8.9)	18 (6.9)	<0.001

Data are in mean (SD) or n (%) as appropriate.

ESS = Epworth Sleepiness Scale; ISI = Insomnia Sleepiness Index; LDL = low-density lipoprotein.

^†^missing cases, n = 230

^‡^missing cases, n = 219

**Fig 1:**
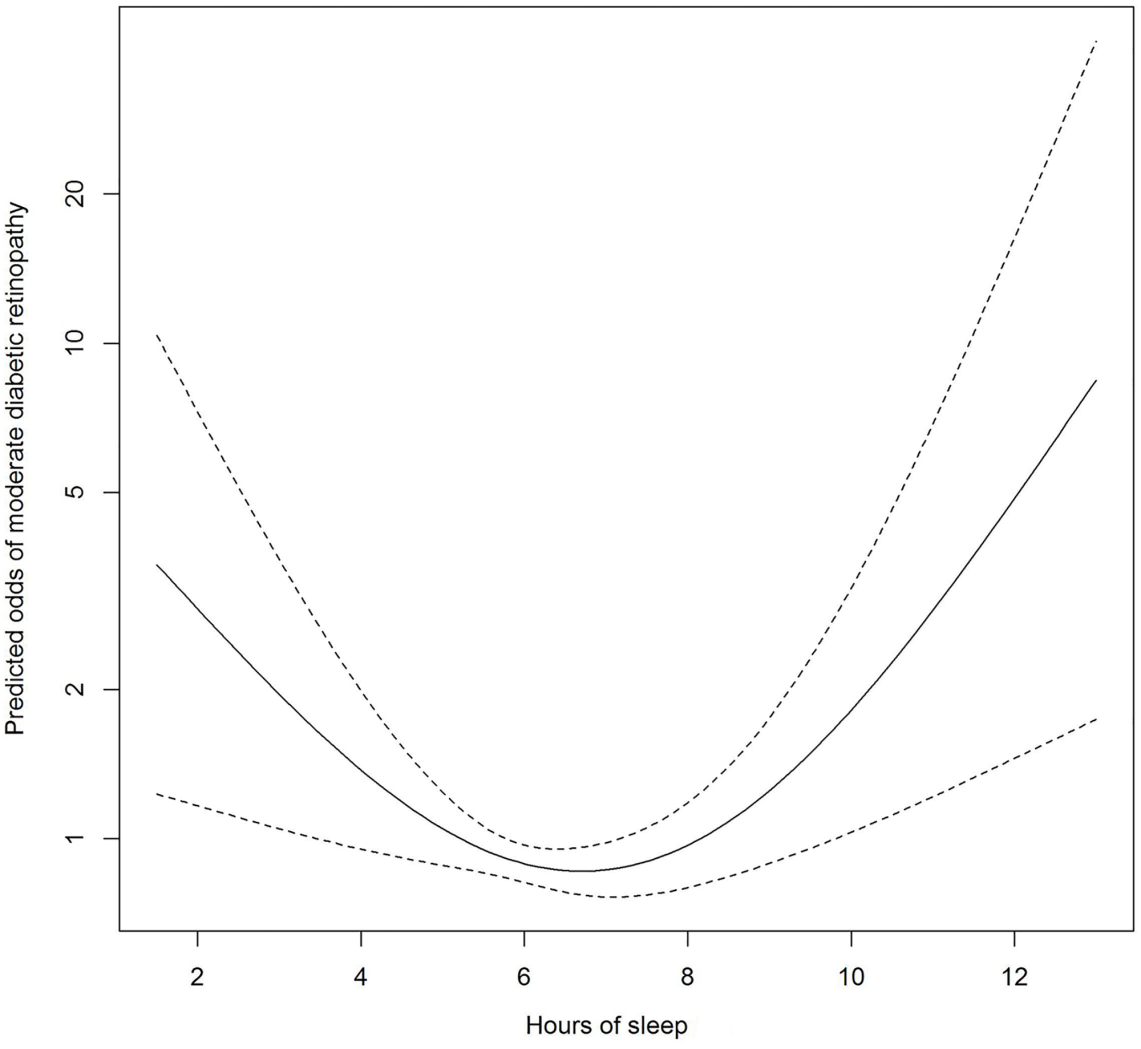
Multivariable-adjusted odds of moderate diabetic retinopathy according to sleep duration. The solid line represents the predicted odds of moderate diabetic retinopathy from non-parametric logistic regression; the dashed lines represent 95% confidence limits. The nonparametric logistic regression was adjusted for age, gender, ethnicity, education, antihypertensive medication, current smoking, respiratory disorders, mood-related complaints, obesity, systolic and diastolic blood pressure, HbA1c, total cholesterol, low-density lipoprotein cholesterol, duration of diabetes, insulin use.

Table 2 shows the associations of sleep duration and other sleep variables with moderate DR. In all models, both short and long durations of sleep were associated with moderate DR. In the multivariable model 2 adjusted for potential confounders, compared to normal sleep duration, short sleep duration had 1.86 increased odds of moderate DR (95% confidence interval [CI]): 1.14–3.00), and long sleep duration had 2.03 increased odds of moderate DR (95% CI: 1.24–3.29). After additionally adjusting for duration of diabetes and use of insulin injections in model 3, these positive association remained significant. In a non-parametric multivariable model examining the dose-response relationship between moderate DR and sleep duration, a U-shaped association was observed between sleep duration and moderate DR (Fig 1). Accordingly, sleep duration was found to follow a quadratic relationship with moderate DR on multivariable polynomial regression (P = 0.01, adjusted for the variables in model 3; table not shown). Table 2 also shows higher ISI scores were also found to be associated with higher odds of moderate DR in models 1 and 2, although this was not significant in model 3. Neither excessive daytime sleepiness nor high risk of OSA were associated with moderate DR in the multivariable analyses.

**Table 2:** Associations of sleep variables with moderate diabetic retinopathy.

		Model 1		Model 2		Model 3*	
Variable	Moderate DR, n (%)	OR (95% CI)	P	OR (95% CI)	P	OR (95% CI)	P
**Sleep duration**							
<6 h (*n* = 294)	40 (13.6)	1.83 (1.17, 2.82)	0.01	1.86 (1.14, 3.00)	0.01	1.73 (1.03, 2.89)	0.04
6≥ h >8 (*n* = 676)	53 (7.8)	Reference		Reference		Reference	
≥8 h (*n* = 261)	36 (13.8)	1.91 (1.21, 3.00)	<0.01	2.03 (1.24, 3.29)	<0.01	2.17 (1.28, 3.66)	<0.01
**Excessive daytime sleepiness**							
Yes† (*n* = 83)	8 (9.6)	0.91 (0.39, 1.82)	0.8	1.26 (0.52, 2.69)	0.57	1.20 (0.47, 2.70)	0.69
No (*n* = 1148)	121 (10.5)	Reference		Reference		Reference	
ESS score, per unit increase		0.98 (0.93, 1.03)	0.52	1.00 (0.94, 1.05)	0.95	0.99 (0.94, 1.05)	0.86
**Obstructive sleep apnea**							
High risk‡ (*n* = 609)	70 (11.5)	1.54 (1.03, 2.33)	0.04	1.29 (0.81, 2.08)	0.29	1.17 (0.71, 1.95)	0.54
Low risk (*n* = 622)	59 (9.5)	Reference		Reference		Reference	
STOP-Bang score, per unit increase		1.11 (0.93, 1.32)	0.25	0.94 (0.75, 1.16)	0.57	0.87 (0.69, 1.10)	0.26
**Insomnia**							
High risk§ (*n* = 192)	29 (15.1)	1.61 (1.01, 2.49)	0.04	1.54 (0.93, 2.51)	0.09	1.37 (0.80, 2.30)	0.24
Low risk (*n* = 1039)	100 (9.6)	Reference		Reference		Reference	
ISI score, per unit increase		1.05 (1.01, 1.09)	0.01	1.05 (1.00, 1.10)	0.03	1.03 (0.98, 1.09)	0.19

ESS = Epworth Sleepiness Scale; ISI = Insomnia Sleepiness Index.

Model 1 adjusts for: age, gender, ethnicity.

Model 2 adjusts for: Variables in Model 1 + education, antihypertensive medication, current smoking, respiratory disorders, mood-related complaints, obesity, systolic and diastolic blood pressure, HbA1c, total cholesterol, low-density lipoprotein cholesterol.

Model 3 adjusts for: Variables in Model 2 + duration of diabetes, insulin use.

*n = 1,001 after excluding cases with missing data on either duration of diabetes or insulin use.

^†^Excessive daytime sleepiness was defined as ≥11 on the Epworth Sleepiness Scale.

^‡^High risk of sleep apnea was defined as ≥3 on the STOP-Bang Questionnaire.

^§^High risk of insomnia was defined as ≥8 on the Insomnia Severity Index.

Table 3 shows the associations between durations of sleep and moderate DR after stratification by ethnicity. In both Malay and Indian groups, the positive associations between both short and long durations of sleep with moderate DR remained consistent in direction in all models, although this was no longer statistically significant in Malays for short sleep duration (model 2, OR [95%]: 1.25 [0.50–2.97]) and Indians for long sleep duration (model 2, OR [95%]: 1.62 [0.86–2.99]).

**Table 3:** Associations of sleep variables with moderate diabetic retinopathy, stratified by ethnicity.

		Model 1		Model 2		Model 3*	
Variable	Moderate DR, n (%)	OR (95% CI)	P	OR (95% CI)	P	OR (95% CI)	P
**Malay Participants Only**							
**Sleep duration**							
<6 h (*n* = 98)	10 (10.2)	1.19 (0.51, 2.64)	0.67	1.25 (0.50, 2.97)	0.63	1.11 (0.43, 2.76)	0.83
6≥ h >8 (*n* = 227)	19 (8.4)	Reference		Reference		Reference	
≥8 h (*n* = 70)	13 (18.6)	2.39 (1.08, 5.14)	0.03	2.98 (1.25, 7.07)	0.01	3.55 (1.43, 8.93)	0.01
**Indian Participants Only**							
**Sleep duration**							
<6 h (*n* = 196)	30 (15.3)	2.21 (1.30, 3.73)	<0.01	2.33 (1.27, 4.24)	0.01	2.35 (1.21, 4.56)	0.01
6≥ h >8 (*n* = 449)	34 (7.6)	Reference		Reference		Reference	
≥8 h (*n* = 191)	23 (12.0)	1.68 (0.95, 2.93)	0.07	1.62 (0.86, 2.99)	0.13	1.49 (0.74, 2.93)	0.26

Model 1 adjusts for: age, gender.

Model 2 adjusts for: Model 1 + education, antihypertensive medication, current smoking, respiratory disorders, mood-related complaints, obesity, systolic and diastolic blood pressure, HbA1c, total cholesterol, low-density lipoprotein cholesterol.

Model 3 adjusts for: Model 2 + duration of diabetes, insulin use.

*n = 1,001 after excluding cases with missing data on either duration of diabetes or insulin use.

**Fig 2:**
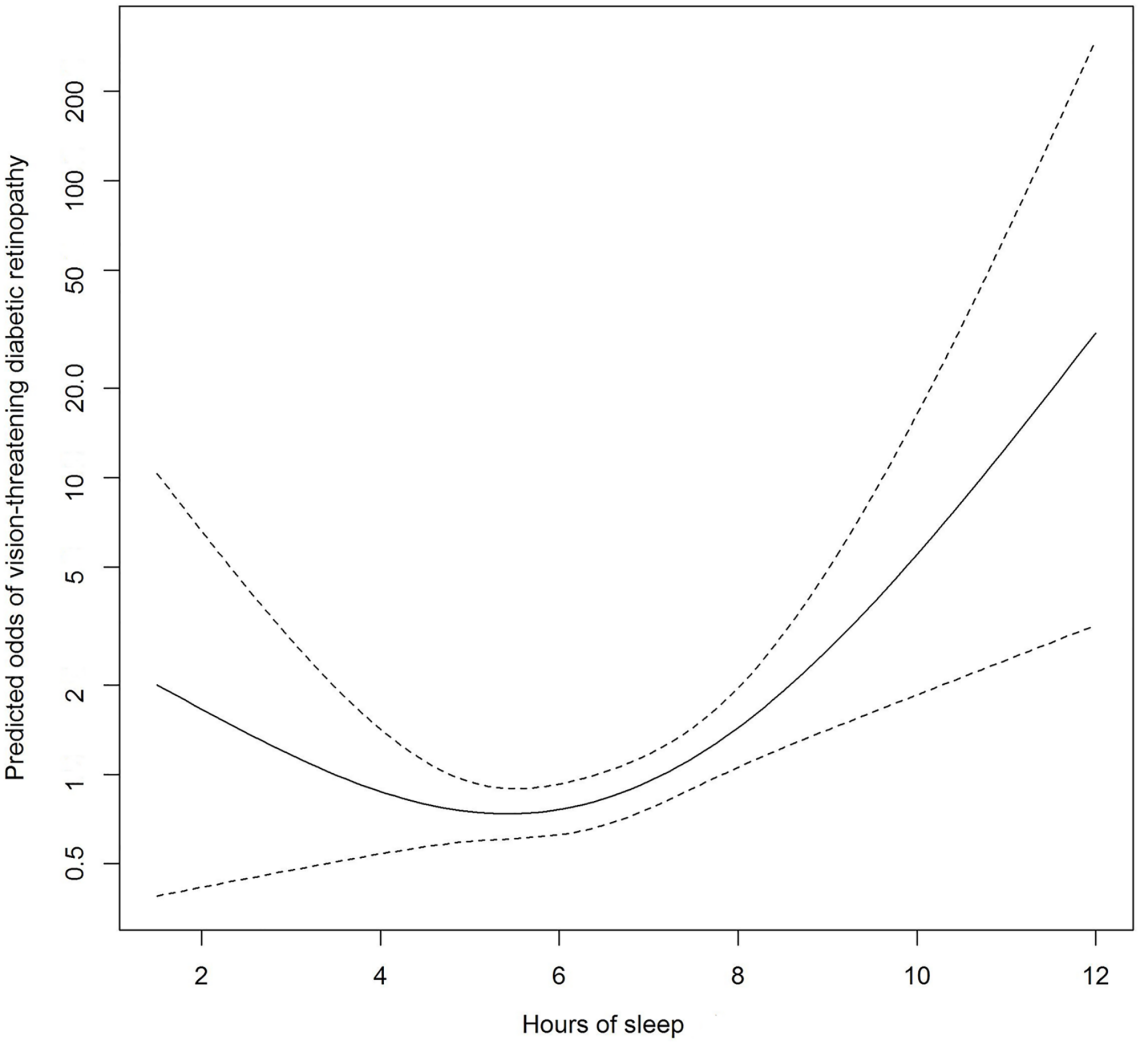
Multivariable-adjusted odds of vision-threatening diabetic retinopathy according to sleep duration. The solid line represents the predicted odds of vision-threatening diabetic retinopathy from non-parametric logistic regression; the dashed lines represent 95% confidence limits. The nonparametric logistic regression was adjusted for age, gender, ethnicity, education, antihypertensive medication, current smoking, respiratory disorders, mood-related complaints, obesity, systolic and diastolic blood pressure, HbA1c, total cholesterol, low-density lipoprotein cholesterol, duration of diabetes, insulin use.

Table 4 shows the associations of sleep duration and other sleep variables with VTDR. In the multivariable model 2, long duration of sleep (OR [95% CI]: 2.06 [1.05–4.04]), excessive daytime sleepiness (OR [95% CI]: 3.15 [1.02–9.34]), and high risk for OSA (OR [95% CI]: 2.25 [1.13–4.60]) were all positively associated with VTDR. In model 3, these positive associations all remained significant, with higher ESS scores also becoming significant. Neither excessive daytime sleepiness nor high risk for OSA were significantly associated with sleep duration (all *P* ≥ 0.35). In supplementary analyses, in addition to the variables in model 3, sleep duration and excessive daytime sleepiness (or high risk for OSA) were adjusted for each other. Including both long sleep duration (OR: 2.43 [1.18–5.05], *P* = 0.02) and excessive daytime sleepiness (OR: 3.43 [1.06–10.93], *P* = 0.04) into the same model, both factors were still positively associated with VTDR. Similarly, including both long sleep duration (OR: 2.61 [1.26–5.49], *P* = 0.01) and high risk for OSA (OR: 2.53 [1.20–5.56], *P* = 0.02) into the same model, both factors were still positively associated with VTDR. Fig 2 shows the dose-response relationship between sleep duration and VTDR using nonparametric multivariable models (adjusted for the variables in model 3) without linearity assumptions. There was an increase in the odds of VTDR with longer durations of sleep; however, the increase in odds of VTDR with shorter durations of sleep was marginal.

**Table 4:** Associations of sleep variables with vision-threatening diabetic retinopathy.

		Model 1		Model 2		Model 3*	
Variable	VTDR, n (%)	OR (95% CI)	P	OR (95% CI)	P	OR (95% CI)	P
**Sleep duration**							
<6 h (*n* = 97)	16 (16.49)	0.73 (0.37,1.39)	0.34	0.74 (0.35,1.52)	0.42	0.78 (0.36,1.65)	0.52
6≥ h >8 (*n* = 165)	35 (21.2)	Reference		Reference		Reference	
≥8 h (*n* = 75)	26 (34.67)	2.02 (1.09,3.73)	0.03	2.06 (1.05,4.04)	0.03	2.37 (1.16,4.89)	0.02
**Excessive daytime sleepiness**							
Yes† (*n* = 19)	7 (36.84)	2.41 (0.86,6.36)	0.08	3.15 (1.02,9.34)	0.04	3.27 (1.02,10.30)	0.04
No (*n* = 318)	70 (22.01)	Reference		Reference		Reference	
ESS score, per unit increase		1.08 (1.01,1.16)	0.03	1.07 (0.99,1.16)	0.07	1.09 (1.01,1.18)	0.02
**Obstructive sleep apnea**							
High risk‡ (*n* = 183)	45 (24.59)	1.86 (1.03,3.42)	0.04	2.25 (1.13,4.6)	0.02	2.24 (1.09,4.75)	0.03
Low risk (*n* = 154)	32 (20.78)	Reference		Reference		Reference	
STOP-Bang score, per unit increase		1.02 (0.78,1.32)	0.89	1 (0.73,1.37)	0.98	0.94 (0.66,1.31)	0.7
**Insomnia**							
High risk§ (*n* = 65)	12 (18.46)	0.65 (0.31,1.28)	0.23	0.73 (0.33,1.53)	0.42	0.73 (0.32,1.59)	0.45
Low risk (*n* = 272)	65 (23.9)	Reference		Reference		Reference	
ISI score, per unit increase		0.97 (0.91,1.02)	0.27	0.97 (0.9,1.04)	0.38	0.97 (0.9,1.04)	0.37

ESS = Epworth Sleepiness Scale; ISI = Insomnia Sleepiness Index.

Model 1 adjusts for: age, gender, ethnicity.

Model 2 adjusts for: Model 1 + education, antihypertensive medication, current smoking, respiratory disorders, mood-related complaints, obesity, systolic and diastolic blood pressure, HbA1c, total cholesterol, low-density lipoprotein cholesterol.

Model 3 adjusts for: Model 2 + duration of diabetes, insulin use.

*n = 312 after excluding cases with missing data on either duration of diabetes or insulin use.

^†^Excessive daytime sleepiness was defined as ≥11 on the Epworth Sleepiness Scale.

^‡^High risk of sleep apnea was defined as ≥3 on the STOP-Bang Questionnaire.

^§^High risk of insomnia was defined as ≥8 on the Insomnia Severity Index.

## Discussion

In a population-based sample of Asian adults with diabetes, we report for the first time a U-shaped association of sleep duration with moderate DR, where both short and long durations of sleep were associated with higher odds of moderate DR; the directions of these associations were consistent in both Malay and Indian adults in subgroup analyses stratified by ethnicity. Additionally, long duration of sleep, high risk of OSA, and excessive daytime sleepiness were positively associated with VTDR. This is consistent with prior reports linking OSA with increased severity of DR, but is the first instance where duration of sleep has been associated with severity of DR. Our findings thus suggest that both quantity and quality of sleep may have implications on the presence and severity of DR.

To date, only 3 studies have reported on the association between sleep duration and DR [9–11]. The first of these cross-sectional studies found no associations between short or long sleep duration with DR on multivariate logistic regressions in Indian persons with diabetes [11]. However, many residual confounders were unadjusted for, as only age, duration of diabetes, and HbA1c were included as co-variables (R. Raman, personal communication, 15 April 2017); furthermore, there were only 33 (15.1%) cases of DR available for analysis [11]. In the remaining two studies of 1,670 Korean and 1,220 Chinese adults with diabetes, long, but not short, duration of sleep was associated with higher odds of DR on multivariable analysis [9, 10]. Amongst these studies, only the Korean study examined for severity of DR, where no significant associations were found between sleep duration and VTDR [9]. Reasons for the discrepancy between these results and ours is unclear. However, a few points should be considered. First, the outcome of interest in the three aforementioned studies [9–11] was any-DR, whereas it was moderate DR in our study, which may be more “diabetes-specific” [17]. Second, there is no established consensus on how to define a “normal” or “abnormal” duration of sleep, especially in the context of the putative association between sleep duration and DR; thus, the reference “normal” duration of sleep as defined in the studies differed (5≤ h ≤9 [11], 5< h <9 [9], 6≤ h ≤9, [10]) from 6≤ h <8 in the present study. We chose 6≤ h <8 as the reference in our study for two reasons. First, based on the distribution of sleep duration in our study (about 55% slept within this reference range, and only about 22–24% slept either shorter or longer than this duration); second, this reference range corresponded roughly to the lowest predicted odds of moderate DR and vision-threatening DR in Figs 1 and 2. However, even if a reference range of 5< h <9^10^ was used, all results remained consistent, i.e. long and short durations of sleep were still significantly associated with higher odds of moderate DR, and long durations of sleep was still significantly associated with higher odds of VTDR (table not shown). Reference ranges of 5≤ h ≤9^9^ and 6≤ h ≤9^11^ were untenable due to the low number of cases in the long sleep duration category (n = 29 for the moderate DR analysis).

Comparatively more studies have examined the relationship between poor sleep quality and DR [12, 27–32]. A meta-analysis of eight studies reporting the effect of OSA on overall DR found no significant association between OSA and DR [12]. The influence of OSA on the severity of DR, however, has been more convincing. Majority of cross-sectional and longitudinal studies have reported OSA to have positive associations with the severity or progression of DR [27, 29–32]; although a study by Banerjee et al.[28] showed no associations, they were limited by a very small sample of VTDR cases. These findings are therefore consistent with our results, which showed high risk of OSA or excessive daytime sleepiness to be associated with higher odds of VTDR, but not moderate DR. Furthermore, after adjusting for sleep duration (since poor sleep quality may alter sleep quantity), excessive daytime sleepiness or being at risk of OSA still had positive associations with VTDR.

The mechanisms linking short or long sleep duration with the prevalence and severity of DR are not fully understood, although some postulations can be made. Sleep deprivation may be deleterious with respect to DR as it has been associated with increased ghrelin and leptin levels (which increases hunger and decreases satiety, and tilts the energy balance towards excess [33]), as well as insulin resistance and poorer glycemic control [5, 6]. However, in our study, sleep duration showed no association with HbA1c levels (assessed as either a continuous or categorical variable), which suggests the possibility of another mechanism separate from glycemic control. A pro-inflammatory state induced by sleep deprivation is plausible explanation, as inflammatory biomarkers have also been linked to DR [34, 35]. Conversely, sleep disruption may instead be a consequence of DR. Night-time melatonin levels were shown to be altered [36], and neurodegeneration of melanopsin-expressing retinal ganglion cells have been found [37], in persons and eyes with severe DR respectively. Disruption of the circadian rhythm may also explain the association of long sleep duration with VTDR. Additionally, long sleep duration may be promote retinal hypoxia as during the dark, rod photoreceptors consume large amounts of energy through the maintenance of dark currents, which increases the retinal oxygen tension [38].

With respect to the association of high risk of OSA or excessive daytime sleepiness with VTDR, our results should be viewed with circumspection since they were based on questionnaires; however, they are in concordance with other studies that have linked OSA, that was measured objectively, to the severity or progression of DR [27, 29–32]. A few pathogenic mechanisms have been suggested: nocturnal oxygen desaturation may induce the expression of vascular endothelium growth factor [39], which is known to promote DR; recurrent central nervous system arousals in OSA may also increase the sympathetic drive [40], which has broad downstream effects, including the predisposition towards hypertension, and the dysregulation of glucose metabolism, which are both harmful in DR.

Alternatively, it is possible that disturbances to the quantity or quality of sleep may have effects on DR mediated through behavioural mechanisms. For instance, sleep restriction may decrease exercise and increase sedentary behaviour [41], which may negatively impact diabetic control. Subjects with poorer quality sleep may also have poorer self-care, dietary adherence and diabetes control [42]. Alternatively, reverse causality may also contribute, since subjects with poorly controlled diabetes may also be affected by neuropathic pain and polyuria that may disrupt sleep. Thus, a limitation of our study is that we were not able to completely control for all such potential confounders (e.g. physical activity, nocturia). Other limitations include the potential misclassification bias of self-reported habitual sleep duration [43], the lack of objective measures for evaluating both the quantity and quality of sleep, and a lack of information regarding the usage of hypnotic medications and continuous positive airway pressure. Lastly, our cross-sectional study design also restricts inferences of causality. Strengths of our study include a large number of participants with diabetes (*n* = 1,231) from two independent population-based studies with high participation rates (72.1–75.5%), the same standardized protocol for grading of DR based on established population-based studies [15], and a standardized assessment of systemic and ocular parameters.

In summary, we have reported novel positive associations between short or long durations of sleep with moderate DR, and between long sleep duration and VTDR. Additionally, we have also found positive associations between high risk of OSA or excessive daytime sleepiness with VTDR, which is consistent with prior reports. These results add to the accumulating evidence that the quantity and quality of sleep may be implicated in DR, amongst other micro- and macro-vascular complications of diabetes [10].
